# Guidelines on the use of sex and gender in cardiovascular research

**DOI:** 10.1152/ajpheart.00535.2023

**Published:** 2023-11-24

**Authors:** Charlotte W. Usselman, Merry L. Lindsey, Austin T. Robinson, Beth A. Habecker, Chloe E. Taylor, W. David Merryman, Derek Kimmerly, Jeffrey R. Bender, Judith G. Regensteiner, Kerrie L. Moreau, Louise Pilote, Megan M. Wenner, Myles O’Brien, Timur O. Yarovinsky, Nina S. Stachenfeld, Nisha Charkoudian, Quin E. Denfeld, Jesse D. Moreira-Bouchard, W. Glen Pyle, Kristine Y. DeLeon-Pennell

**Affiliations:** ^1^Cardiovascular Health and Autonomic Regulation Laboratory, Department of Kinesiology and Physical Education, McGill University, Montreal, Quebec, Canada; ^2^School of Graduate Studies, Meharry Medical College, Nashville, Tennessee, United States; ^3^Research Service, Nashville Veterans Affairs Medical Center, Nashville, Tennessee, United States; ^4^Neurovascular Physiology Laboratory, School of Kinesiology, Auburn University, Auburn, Alabama, United States; ^5^Department of Chemical Physiology and Biochemistry and Knight Cardiovascular Institute, Oregon Health and Science University, Portland, Oregon, United States; ^6^School of Health Sciences, Western Sydney University, Sydney, New South Wales, Australia; ^7^Department of Biomedical Engineering, Vanderbilt University, Nashville, Tennessee, United States; ^8^Autonomic Cardiovascular Control and Exercise Laboratory, Division of Kinesiology, School of Health and Human Performance, Faculty of Health, Dalhousie University, Halifax, Nova Scotia, Canada; ^9^Section of Cardiovascular Medicine, Department of Internal Medicine, Yale Cardiovascular Research Center, New Haven, Connecticut, United States; ^10^Department of Immunobiology, Yale University School of Medicine, New Haven, Connecticut, United States; ^11^Divisions of General Internal Medicine and Cardiology, Department of Medicine, Ludeman Family Center for Women’s Health Research, University of Colorado Anschutz Medical Campus, Aurora, Colorado, United States; ^12^Division of Geriatrics, Department of Medicine, University of Colorado Anschutz Medical Campus, Aurora, Colorado, United States; ^13^Eastern Colorado Health Care System, Geriatric Research Education and Clinical Center, Aurora, Colorado, United States; ^14^Centre for Outcomes Research and Evaluation, Research Institute of the McGill University Health Centre, McGill University, Montreal, Quebec, Canada; ^15^Department of Kinesiology and Applied Physiology, University of Delaware, Newark, Delaware, United States; ^16^School of Physiotherapy and Department of Medicine, Faculty of Health, Dalhousie University, Halifax, Nova Scotia, Canada; ^17^John B. Pierce Laboratory, New Haven, Connecticut, United States; ^18^Department of Obstetrics, Gynecology and Reproductive Sciences, Yale School of Medicine, New Haven, Connecticut, United States; ^19^Thermal and Mountain Medicine Division, United States Army Research Institute of Environmental Medicine, Natick, Massachusetts, United States; ^20^School of Nursing and Knight Cardiovascular Institute, Oregon Health and Science University, Portland, Oregon, United States; ^21^Q.U.E.E.R. Lab, Programs in Human Physiology, Department of Health Sciences, Boston University College of Health and Rehabilitation Sciences: Sargent College, Boston, Massachusetts, United States; ^22^IMPART Team Canada Network, Dalhousie Medicine, Saint John, New Brunswick, Canada; ^23^Department of Biomedical Sciences, University of Guelph, Guelph, Ontario, Canada; ^24^School of Medicine, Division of Cardiology, Department of Medicine, Medical University of South Carolina, Charleston, South Carolina, United States; ^25^Research Service, Ralph H. Johnson Veterans Affairs Medical Center, Charleston, South Carolina, United States

**Keywords:** cardiac, gender diversity, rigor and reproducibility, vascular

## Abstract

In cardiovascular research, sex and gender have not typically been considered in research design and reporting until recently. This has resulted in clinical research findings from which not only all women, but also gender-diverse individuals have been excluded. The resulting dearth of data has led to a lack of sex- and gender-specific clinical guidelines and raises serious questions about evidence-based care. Basic research has also excluded considerations of sex. Including sex and/or gender as research variables not only has the potential to improve the health of society overall now, but it also provides a foundation of knowledge on which to build future advances. The goal of this guidelines article is to provide advice on best practices to include sex and gender considerations in study design, as well as data collection, analysis, and interpretation to optimally establish rigor and reproducibility needed to inform clinical decision-making and improve outcomes. In cardiovascular physiology, incorporating sex and gender is a necessary component when optimally designing and executing research plans. The guidelines serve as the first guidance on how to include sex and gender in cardiovascular research. We provide here a beginning path toward achieving this goal and improve the ability of the research community to interpret results through a sex and gender lens to enable comparison across studies and laboratories, resulting in better health for all.

Listen to this article’s corresponding podcast at https://ajpheart.podbean.com/e/guidelines-on-use-of-sex-and-gender-in-cardiovascular-research/.

## Graphical abstract

**Figure:**
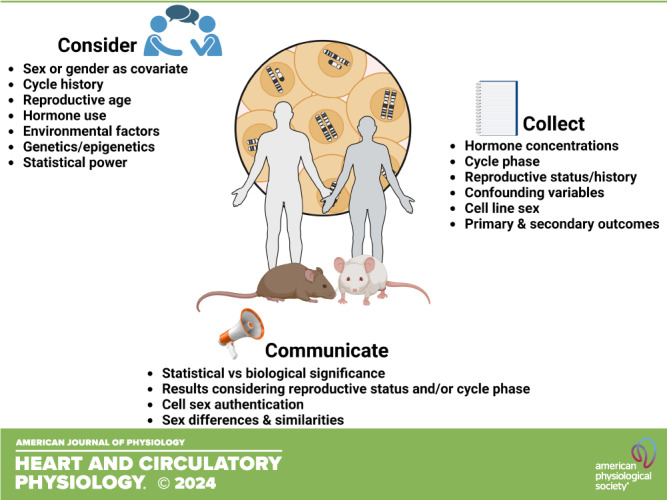

## INTRODUCTION

Until as recently as the early 1990s, women were generally excluded from research studies because of the preconceived and untested notion that sex hormone concentrations in women fluctuated to such an extent that experimental results would be highly variable, resulting in uninterpretable data. In addition, there was the perception that research could pose risk to women of childbearing potential (1, 2). It was also believed that results from men could simply be generalized to women, a concept that is not evidence based and which is potentially dangerous to women and gender-diverse individuals, particularly those from diverse ethnic and racial groups (3). The United States Food and Drug Administration found that among all medications withdrawn from market between 1997 and 2000, more were withdrawn due to harm incurred to women than to men (3, 4). In addition, as a result of the lack of focus on sex and gender in human research, there are currently no sex-specific clinical care guidelines (4). This underscores the importance of understanding and studying sex and gender differences and the need to implement effective and specific strategies for the prevention and treatment of cardiovascular diseases for all people.

Although sex and gender are terms often used interchangeably, sex refers to biological attributes (e.g., chromosomes, gonadal organs, and genetics) whereas gender refers to socially, psychologically, and culturally constructed factors that shape behaviors, stereotypes, and attitudes across societies over time (5–8). In genome-wide association studies, the autosomal signals are often studied, and the sex chromosomes are not considered. In addition, sex stratification is not always performed and only adjustments are accounted for in regression models (9).

Sex exists on a spectrum, and includes those who are male and female as well as those who are intersex. Gender also exists on a spectrum, and includes men, women, nonbinary people, and agender people, among additional identities (6). Where sex may influence the biological differences and effects to a medication or intervention in an individual, gender encompasses a wider spectrum of factors including behavior and social interactions (10, 11). Often, sex is also associated with disease outcome(s). For example, despite being more likely to seek medical care, women receive treatments for similar complaints to men less often than men do, because of clinician implicit bias, thus affecting outcome(s) (11, 12). Sex and gender influences are also found in clinical care experiences as women are more likely to be misdiagnosed or have symptomology ignored, and are less often prescribed medications and procedures known to have beneficial effects in such diseases as diabetes and heart disease even when clearly indicated (13). Socioeconomic forces must also be considered because women and gender-diverse people are more likely to experience poverty (14). Socioeconomic factors also have implications for nutritional differences, which can translate to differences in the gut microbiome. The gut microbiome is increasingly recognized as an important modifier for sex and gender differences (15).

As a point of clarification, because of the lack of standardized definitions for the guidelines, we use woman to include any individual who considers themselves to be a woman. The same consideration is given when referring to men. Because prior studies have not always defined terms within their articles, in general the assumption can be made that the term women refers to cis-gender women (i.e., women whose gender is consistent with their sex assigned at birth) and the term men refers to cis-gender men (i.e., men whose gender is consistent with their sex assigned at birth). We recommend in the future that these terms should be more clearly defined in methods, results, and discussion sections (8, 16). Investigators should clearly state terminology used (women/men or female/male) and should be consistent throughout the paper as to not conflate sex and gender, and also to state if transgender individuals are included within the participant groups. For transgender adults using hormone therapy, we refer the reader to published guidelines from the American Heart Association (17).

Since 1993, the National Institutes of Health (NIH) has implemented several initiatives and mandates to include women in research. These initiatives include attempts to enhance reproducibility through rigor and transparency by requiring researchers to account for sex as a biological variable (SABV) in research design, analysis, and reporting for both human and animal studies. Other government-based international research funding agencies, including the Canadian Institutes of Health (CIHR) and the European Commission (EC) also require that researchers integrate sex and gender into biomedical research (1, 18, 19).

By 2016, it had become standard to consider sex and gender for human studies, although this standard mainly pertained to binary sex, excluding intersex and gender-diverse individuals. Furthermore, research using cells and animal models had not begun considering SABV. Thus, in 2016 NIH instituted an initiative to encourage researchers to incorporate the use of SABV for animal studies, including research using cell-based assays, an often-overlooked consideration. The expectation of NIH was that all applicants proposing animal studies would factor SABV into their experimental design, data analysis, and reporting—or—provide strong scientific justification for single sex investigation (see NIH NOT-OD-15-102, https://grants.nih.gov/grants/guide/notice-files/not-od-15-102.html). More recently, journals including *American Journal of Physiology-Heart and Circulatory Physiology* (*AJP-Heart and Circ*) have supported NIH efforts by mandating that researchers include and consider SABV and gender in their studies unless there is strong scientific justification for an exception (20, 21). Despite recent improvements in reporting SABV, sex omission, cisgender, heteronormative, male bias, and lack of analyses on how cell sex may affect the results remain widespread (21).

The following guidelines focus on how to incorporate sex, gender, and their interactions on cardiovascular outcomes, and complement other resources published by the *AJP-Heart and Circ* (20, 22, 23). We have confined the scope to include cell- to individual-based research and have not included population research, which has been previously addressed (24–29). Although each section has a particular focus, an underlying and common thread that weaves through each segment is the concept to start simple and build from there.

## INCORPORATING SEX AS A BIOLOGICAL VARIABLE IN HUMAN STUDIES

Common misperceptions raised when human physiology researchers are asked to consider SABV include *1*) sample size would need to be doubled to maintain statistical power when analyzing more than one sex, which increases the time to complete experiments and doubles the budget needed; *2*) women are more difficult to study than men because of hormonal changes associated with the menstrual cycle, hormonal contraceptive use, and the menopause transition (i.e., perimenopause) that increases heterogeneity of response; and *3*) beginning the study with men is acceptable, with the plan of adding women later after the initial data are collected. *Assumptions 1* and *2* have not held up with actual research, and the current statistics on use of more than one sex reveals that rarely do follow-up studies in women actually occur, unless mandated by funding agencies and journals. Therefore, because women make up over half of the human population, it is critical that research studies include both women and men to facilitate a complete understanding of human health and disease. Table 1 provides minimum and ideal recommendations for considering sex in human studies. In the following sections, we discuss several factors (i.e., menstrual cycle, hormonal contraceptive use, gonadal aging) that need to be considered when designing studies.

**Table 1.** Minimum and optimal recommendations for use of sex and gender in human studies

Minimum	Optimal
*Study design, data collection, and data analysis*	
• Include women and men in all human trials unless strong scientific justification. • Include gender nonconforming people as much as possible. • May not need to increase sample size. • Report sex-specific or sex-dependent effect sizes when possible. • Include sex and gender as covariate(s) when possible. • Identify gender of all participants and participant-reported sex at birth. • Document details of menstrual cycle- natural vs. hormonal contraceptive (with type, dose, and duration of use). • If possible, estimate and record participant menstrual cycle day. • Collect menopausal status.	Minimum, plus: • Detail menstrual cycle history. • Use ovulation kits to characterize cycle phase. • Sufficiently power study for covariate(s) or split analysis. • Explore equivalence and interaction testing (moderation analysis). • Report dose and type of estrogen and dose and type of progestin for oral contraceptives. • Stratify participants based on hormonal contraception type/dose or include only participants on same type/dose. • Control for menstrual cycle phase if appropriate. • Perform longer-term tracking of phase durations by urine, blood, or plasma markers. • Control for age, time of day (circadian rhythm), exogenous reproductive hormone use, other medication. • Measure estradiol, luteinizing hormone, follicle stimulating hormone, estrone, progestins, SHBG, and androgens.
*Reporting*	
• Report individual male/female data in tables and figures as separate symbols. • Include information regarding biological sex (at birth) and gender identification. • Report hormone contraception use and type. • Report a priori power analysis results if primary focus is on sex differences. • Include data availability statement. • Use The Stages of Reproductive Aging Workshop (STRAW + 10) criteria to characterize menopausal status and reproductive age.	Minimum, plus: • Document self-reported menstrual/oral contraceptive pill phase at time of testing and cycle history when possible. • Report sex hormone concentrations (absolute/relative levels and relevant ratios). • Report individual regularity of natural menstrual cycle status. • Report duration of contraceptive use. • Identify duration of hormone therapy. • Report pregnancies and births. • Report effect sizes for sex comparisons (e.g., Cohen’s *d*, correlations, and odds ratios). • Explore potential confounding variables through sensitivity analyses (e.g., body composition).
*Data interpretation*	
• Distinguish statistical vs. clinical/physiological differences. • Include limitations (e.g., confounding factors, sample size). • Keep focus within the context of your sample set.	Minimum, plus: • Include interpretation of data based on menstrual cycle/phase, contraceptive use, or menopausal status as investigated/ appropriate.

### Considerations for Study Design

#### Inclusion of women, aging, and health.

Women make up over half of the human population, so it is critical to be inclusive in research studies to enable a complete understanding of human health and disease. Women should prospectively be incorporated into cardiovascular research studies (30, 31). In general, efforts should be made to include roughly equal numbers of women and men into a study while taking into consideration sex-dependent factors that may affect the research question (32). For some but not all cases, there may be a need to increase sample size to attain adequate power to detect sex differences (33, 34). However, depending on the context, multiple factors must be taken into consideration when deciding on the sex breakdown of the sample size (35). Another consideration is whether the study is focused on normal cardiovascular physiology within the general population. If the focus is on the general population, further points to consider include the age of the participants and their reproductive stages and menstrual cycles. For example, women and men age differently (e.g., menopause vs. gradual declines in testosterone), which impacts physiological sex differences and how we study them. Broadening the age range of inclusion may also be necessary to account for sex-age interactions. Importantly, not including enough women in a study will ensure that sex and/or gender-specific results are not obtained. In this context, the Systolic Blood Pressure Intervention Trial (SPRINT-2015) reported that intensive blood pressure targets were associated with 27% lower all-cause mortality than the standardized targets (systolic blood pressure, 140 mmHg). This trial was expected to lead to sex-specific guidelines (16, 36), however, only 30% of SPRINT participants were women, and the follow-up time was reduced in women. Sex-specific guidelines did not result, in part because of a lack of statistical significance.

If a study focus is on a particular disease condition (e.g., heart failure or ventricular tachyarrhythmias), then other points to consider include sex differences in disease prevalence, as well as the interaction with age (i.e., are there differences in disease incidence across the life span, such as in coronary heart disease or heart failure; 37). As such, efforts should be made to approximate the prevalence of the disease in the broader population. Taken together, there are powerful sex-specific covariates that may influence one sex versus the other. As such, it is important to also consider the interplay of these covariates and ensure that they are considered in the study design. Researchers can also consider whether a disease has been understudied in one sex or oversampled in a particular sex.

#### Menstrual cycle.

Although some investigators suggest that control of the menstrual cycle is a requirement of a strong study design for evaluating cardiovascular control in women (38), others counter that such consideration is not always necessary depending on the study design and central research questions (39). Nonetheless, because sex hormones impact cardiovascular function, menstrual cycle phase should be considered when designing and conducting research studies (38, 40–42).

The menstrual cycle can be divided into three primary phases: the follicular phase, ovulation, and the luteal phase. The average menstrual cycle length is ∼28 days (43), with the onset of menstruation occurring on *day 1* and ovulation by *day 14* of the menstrual cycle (44). Because of the fluctuations in estrogens and progesterone across the menstrual cycle, researchers have typically conducted studies during either the early follicular phase (*days 1–7* when both estradiol and progesterone are low), and/or preovulatory/late-follicular (when estradiol is high, but progesterone is low), and/or the midluteal phase (∼10 days following ovulation when both estradiol and progesterone are high). For example, in studies that examine sex differences, one common approach that has been used to control for the menstrual cycle includes testing women during the early follicular phase of the menstrual cycle to minimize the potential influence of sex hormones on outcomes of interest. Although this is the most convenient phase to characterize (because it is easy to identify *day 1* of menstruation and count forward), it is important to keep in mind that sex hormone concentrations are still different between women and men (e.g., ∼10- to 15-fold higher testosterone levels in men compared with women; 41). Moreover, women spend ∼25% of their reproductive lives during this phase, so obtaining information only during this phase limits our understanding of the physiology of women and of the impact of changes in estradiol and progesterone on physiological outcomes (40, 41). A lack of sex difference when women are tested during this phase, therefore, may not remain when hormones are elevated.

If one is interested in targeting specific phases of the menstrual cycle to understand the impact of fluctuations in sex hormones, it is important to note that the “normal” 28-day menstrual cycle may vary substantially in young, healthy women (23–36 days), and ovulation does not always occur on *day 14* (44). Depending on cycle length, women can ovulate on *days* ∼*10*–*17* or not at all (anovulation). For these reasons, if the goal is to evaluate the influences of endogenously cycling sex hormones in women, researchers should ideally use some combination of ovulation predictor kits and blood sampling to predict and subsequently confirm menstrual cycle phases. The latter is important because even with menstrual cycles that are regular, there is interindividual variability in the circulating hormone concentrations.

We must recognize that the impact of sex hormones can be as powerful as other variables typically controlled for in experiments, such as time of day, medications, fasting status, posture, prior exercise, caffeine use, room temperature, or hydration. However, when considering SABV, it is sometimes the case that testing women during a specific phase of the menstrual cycle is not possible, as with certain large-scale clinical trials, those with complex study designs, or field research. Where possible, we recommend controlling for or, at a minimum, reporting menstrual cycle phase, as described earlier.

#### Hormonal contraceptives.

Over 90% of women use hormonal contraception at some point in their lives. As such, it is important to investigate the impact of hormonal contraceptives on physiological variables, and include methods (i.e., hormonal intrauterine devices, the ring, patch, and injectables) beyond oral contraception. In addition, many women use hormonal contraceptives to treat syndromes including polycystic ovary syndrome (45, 46), psychiatric disorders (47), endometriosis (48), and premenstrual and perimenopausal symptoms (49). Some progestins have mild androgenic properties relative to endogenous progesterone (50) and can alter peripheral circulation (51, 52), blood pressure (53), cardiac contractility (54), as well as aldosterone function (55) and release (56). How these conditions and components interact to influence cardiovascular responses is understudied.

Hormonal contraceptives contain either progestin or combined progestin and an estrogen and work by preventing ovulation or by thickening cervical mucus, or both, and some also act as spermicides. Hormonal contraceptives can be in the form of oral pills, patches, rings, or intrauterine devices. It is important for researchers to account for and record the type/dose of contraceptive, and use caution with collapsing all contraceptive users into one group given differences in hormone type/dose across various generations of pills. For additional information on this topic and tips for study design, we refer the reader two excellent reviews (57, 58). Finally, very few studies have examined the impact of the progestogen intrauterine devices on the cardiovascular system and given the increase in popularity of this type of contraceptive, it is important for future studies to determine any acute and/or chronic impacts on cardiovascular function.

#### Gonadal aging in women.

The aging process is another factor that has notable sex differences in that gonadal failure (i.e., menopause), and decline in sex hormones is an inevitable event that occurs around midlife in women but is rare in men until much later in life. In addition, although menopause is often thought as a single event in time, the transition phase leading up to menopause (i.e., perimenopause) is characterized by irregular changes in menstrual cycle length, variable estradiol, and progesterone concentrations while luteinizing hormone (LH) and follicle stimulating hormone (FSH) levels increase, as well as changes in bleeding patterns (sometimes very heavy). There can also be a change in androgen production, impacting the estrogen to androgen ratio (59). Moreover, the menopause transition can also be associated with symptoms that can impact cardiovascular physiology, including, vasomotor (i.e., hot flashes, night sweats), sleep disturbances, and anxiety and depression.

The Stages of Reproductive Aging Workshop (STRAW + 10) criteria were written and developed to characterize and describe phases of reproductive aging (60). We recommend using this resource to characterize midlife women who are transitioning through menopause, and that investigators document not only chronological age but also reproductive age. For example, self-report questions on age at the onset of menopause, type of menopause (e.g., natural, surgical, hysterectomy, oophorectomy), or cancers that caused early menopause can be useful. Hormonal contraceptive use, most recently hormonal intrauterine devices, have increased in midlife women to control bleeding, and to prevent pregnancy. We also recommend analyzing blood for LH and FSH. Because late perimenopausal women have infrequent cycles, it may not be possible to test them during a specific phase. Therefore, we recommend testing at a time of convenience to the participant and obtaining a detailed cycle history and blood sampling for analysis of sex hormones (e.g., estradiol, estrone, progesterone, sex hormone-binding globulin (SHBG), LH, FSH, and androgens).

#### Sex hormones in men.

Although not as drastic as the influence of the menstrual cycle or gonadal aging in women, there is considerable intraindividual variability of testosterone in men, driven by many factors including energy balance, sleep, illness, circadian rhythm, age, and body composition (61–64). In addition, there is considerable interindividual variability in testosterone levels in men, even when controlling for age (65, 66). Testosterone exhibits diurnal and day-to-day variability in men, which is often not accounted for in research studies. In contrast to the rapid decrease in estradiol with menopause, men demonstrate a gradual decline (∼1%/year) in testosterone after the third decade (65, 66). Importantly, 10–15% of men in their 50s, and 50% of men over 80 yr of age, exhibit circulating total testosterone concentrations below the normal range for young men (67, 68). Thus, because testosterone (and estrogen) can regulate cardiovascular physiology in men (69–75), it is just as important to measure sex hormones in men as it is in women, particularly in aging research.

#### Additional considerations.

Another important consideration for studies focused on sex hormones as a primary outcome, irrespective of biological sex of the participants, is that the majority of circulating testosterone is tightly bound to SHBG. If one is seeking to quantify bioavailable hormones (e.g., testosterone, estrogen), measuring free concentrations of the hormone, the fraction that is either free or loosely bound to other proteins, primarily albumin, is required (see Table 1). Finally, we have mostly discussed estrogen and progesterone in the context of female physiology and testosterone in male physiology, however, there is little data examining the impact of testosterone on the female system in humans. For example, women with chronic androgen excess polycystic ovary syndrome (AE-PCOS) and transgender men receiving gender-affirming hormone therapy (GAHT) are two conditions in which a female vasculature is chronically exposed to androgens. These high androgen exposures are both associated with changes in cardiovascular physiology (76–79). It is unclear whether these cardiovascular risks increase as women with AE-PCOS or transgender men age. It is a significant area of inquiry because changes in cardiovascular and metabolic physiology become apparent when exposed to androgens and the vascular system in women is generally considered protected by high levels of estrogens.

### Considerations for Data Collection

Recruitment of human research volunteers is often challenging, especially meeting target numbers of both women and men sufficient for powering the study. Thus, different strategies are needed to ensure that studies are on track to enroll prespecified numbers of women and men throughout the study (80). First, it is important to track the numbers of women and men enrolled from the beginning of the study as this will help reduce potential bias. Second, recruitment strategies need to consider sex/gender-dependent factors that would imbalance the approach to recruitment. For example, ensuring that recruitment fliers are placed in locations frequented by both women and men, such as gyms, churches, restaurants, shops, and community centers. When recruiting from clinical locations (e.g., cardiology clinics), it is important to consider factors (e.g., type of clinic, balance of women vs. men providers) that may bias against one sex versus another. For example, women are less likely than men to be referred to a cardiologist or specialty cardiologist (81), which in turn would limit the number of women available to approach for participation in the study. One suggestion to ameliorate this potential bias is to expand recruitment to additional clinical locations, particularly ones that have a larger patient pool. Third, when discussing the study with potential participants, consider sex/gender-dependent barriers that would influence whether a particular person is able to participate. Considering solutions to these barriers during experimental design will help to reduce the influence of barriers. For example, women are more often informal caregivers for others (both young and old); these family commitments may preclude a woman’s ability to commit to research studies (82). Thus, providing childcare during participation may need to be included in trial design, or offering research slots outside of normal business hours when other family members may be able to fill the role of caregiver. Researchers should consider strategies at multiple levels to ensure adequate sex and gender balance in research studies (80).

### Considerations for Data Analysis, Reporting, and Interpretation

The analytical approach and corresponding interpretation of sex-specific differences are dependent upon the research question of interest (33). These recommendations were developed to assist cardiovascular researchers with a wide range of experience and research infrastructure.

The degree to which SABV is incorporated into analysis depends on the a priori objective of the study. If not the primary objective of the study, we suggest the following minimum recommendations: *1*) include sex as a covariate to provide insight into whether sex impacted the main outcomes, *2*) report effect sizes for between sex comparisons to aid sample size calculations to help ensure future studies are statistically powered, and *3*) examine sex-disaggregated data to look at signals for differences and also to allow meta-analysis.

At the next level, more thorough approaches to analyzing sex-specific or sex-dependent outcomes can be incorporated such as documentation of menstrual cycle/oral contraceptive pill phases, and/or aging-related outcomes. However, the ability to do this will be dependent on sample size, outcomes/groups of interest (e.g., menopause status and contraceptive use), and the variability of the sex-specific or sex-dependent factors (e.g., sex hormone levels). Sufficiently powered studies may consider examining whether statistically significant interactions exist regarding association with the outcome variable. If so, the sample may be split (e.g., between sexes, menstrual/pill phases, testosterone sufficiency, and menopausal status) and analyses conducted separately for each group. Depending on the heterogeneity of the sample, covarying for impactful within sex-specific variables may be warranted (e.g., estradiol or testosterone concentration, generation of contraceptive pill, and menopausal status). Similarly, sensitivity analyses may be conducted to explore potential pathways, or control for confounding systemic factors (e.g., body composition) and/or sex-impacted plasma markers (e.g., adipokines and steroids).

Statistical methods including *t* test and ANOVA have been implemented to determine the impact of sex. However, an absence of statistical effects (i.e., large *P* value) is not evidence of no impact (i.e., no sex differences; 83). Equivalence testing can determine whether two groups provide statistically equivalent outcomes (i.e., they differ by less than the smallest effect size of interest). Equivalence testing permits the distinction of whether cardiovascular measures are statistically equivalent/nonequivalent between men and women or within a sex when stratified, rather than implying equivalence based on not different values (83). Another approach to identifying sex differences is to use interaction testing (i.e., moderation analysis), which analyzes whether the relationship between two variables differs by a third variable (e.g., sex). For example, one paper showed that the relationship between the left ventricle at end-diastole and heart failure symptoms differed between men (dilation) and women (reduced compliance) (84). However, a limitation of interaction testing is the requirement that a relatively large sample size be investigated. This is why sex-disaggregated data are needed for meta-analysis and sex interaction testing.

At a minimum, reporting the proportion of men and women in the sample, as well as their gender identifications, are needed. Among premenopausal women, it is recommended to document contraceptive use (yes/no, pill type, intrauterine device, duration of use); self-reported indication of regular menstrual cycle status (i.e., ∼28 days); and approximate menstrual/pill phase at the time of testing. In older adults, it is recommended to document their age at menopause (postmenopausal females) and usage of hormone therapies (both sexes). If a priori interests in determining sex differences are specified, it is important to include the initial sample size calculation based on an effect size of interest (e.g., Cohen’s *d*, correlation strength). Recommendations include reporting the dose of estrogens and type and dose of progestin when including women taking hormonal contraception. At present, there is no consensus on the choice of nomenclature (i.e., use of female/male vs. women/men) in cardiovascular studies in humans, given challenges in separating sex- versus gender-driven effects. In general, the terms female/male have been used to present sex-specific findings whereas the terms women/men have been used to comprise gender. We advise that authors include specific details regarding sex and gender, including the collection and reporting of both sex assigned at birth and gender with which you currently identify (see Table 1).

Acknowledging that infrastructure-related barriers exist for many researchers (e.g., cost, storage equipment, and specialized staff), we highlight additional ideal measurements to be reported that exceed the minimal self-report-based information. Specifically, reporting sex-hormone concentrations (e.g., testosterone, estrogen, and progesterone), as well as confirmation of pregnancy status, menstrual, or oral contraceptive pill phase via blood/plasma markers are recommended. When reporting concentrations, both the absolute values and relevant ratios (e.g., estradiol:progesterone ratio) can be reported (85). Tracking menstrual cycle phases across multiple months would ensure a stable menstrual phase duration. Among older adults, a confirmation of menopausal status (women) or lower testosterone (men) via circulating sex hormone concentrations would provide confirmation of sex hormone deficiencies in these populations.

Highlighting results separately for individual participants in figures (e.g., scatterplots) is an important aspect of data visualization regarding whether sex-specific and/or sex-dependent patterns exist, which builds upon recent recommendations of presenting individual data (86). Furthermore, this approach enables other researchers to extract individual data points using semiautomated software (e.g., WebPlotDigitizer) to aid in future sample size calculations (87). Alternatively, including individual data in a repository would be useful to help confirm (or deny) the results of others, or to help conduct detailed subgroup analyses in future larger-scale studies.

The interpretation of results relies largely on statistical testing. The use of magnitude-based inferences (e.g., effect size) provides complementary information to statistical significance in that it determines whether clinical/physiological relevant changes or differences were observed. Study results should be discussed in context of the magnitude of the observed effect and provide an indication of whether clinically meaningful differences were observed. Studies establishing minimal meaningful differences for frequently used experimental measures may be needed. An analysis of interaction is also needed.

Depending on the population under study, results should be interpreted within the confines of the sample included (e.g., patients with heart failure, women in specific menstrual cycle phases, postmenopausal women). For example, sex differences may not be observed when women were tested during the early follicular phase, but present during a higher-estrogen phase (e.g., late-follicular phase). In this example, it would be imperative to emphasize the menstrual cycle phase in the discussion/conclusion sections. When interpreting findings, highlight potential confounding factors (e.g., lifestyle behaviors) that may have impacted study results. Depending on the smallest effect size of interest for comparisons, acknowledge if the study was statistically powered to detect differences in subgroup analyses (e.g., naturally menstruating vs. oral contraceptive pill users).

When investigators are striving for precise control of sex hormone exposure to study a specific variable, gonadal hormone suppression (using a GnRH agonist or antagonist) with selective sex hormone add-back can be used. This requires collaboration and oversight with a clinician familiar with the use of GnRH analogs and/or sex hormone treatments. Although the GnRH agonist/antagonists can be very expensive, less expensive, and less invasive alternatives have recently become available. Details on this methodology can be found in several reviews and original research articles (51, 88–92).

## INCORPORATING GENDER INTO HUMAN STUDIES

In addition to the recommendations provided earlier that have application to incorporating gender, there are other considerations that apply (93).

### Considerations for Study Design

The most critical consideration is to prospectively incorporate gender into cardiovascular research studies rather than try to incorporate retrospectively (30, 31). Although biological sex may affect the likelihood of developing a particular health condition, or responses to interventions, gender may impact the exposure to disease, the likelihood of receiving a treatment, and compliance with interventions and thus disease progression (94). Moreover, gender is diverse and exists along a spectrum that is independent of sex, meaning that the researcher cannot infer gender from participant sex. The idea of accounting for gender differences and gender-related variables is relatively new as previously noted and is evolving currently, as the impacts of gender-related variables are increasingly studied and more clarity is emerging. Accurately capturing the gender identity of each participant enrolled in the study in question through the application of detailed survey demographic questions will ensure that all genders present in the participant sample will be recorded and can be included in statistical analysis (6). Many researchers have found the prospective incorporation of gender as an independent variable particularly challenging, largely because of the lack of standardized definitions and methods. The research question and hypothesis should drive the choice of gender-related variables, considering the psychosocial and behavioral factors that may be influential. It should be noted, however, that complexity of the analysis plan related to identifying gender differences is not a sufficient reason for the exclusion of gender-diverse individuals. Exclusion of those belonging to additional populations beyond cisgender men is what initially led (and continues to lead) to the lack of available knowledge on physiologies in these groups (95).

Gender-related variables can be categorized under the four domains proposed by the Canadian Women’s Health Research Network: gender identity (asked distinctly from sex at birth), gender roles (e.g., caregiver responsibility, primary earner status), gender relations (e.g., marital status, gender-based violence), and institutionalized gender (e.g., wage gap, gendered policies/laws; 11). Such variables have been shown to impact health outcomes (10, 96–99), and it is recommended that a literature review or a priori assessment be performed in the planning stage to identify the most relevant factors to the scientific relationship of interest, while also considering the size and scale of the study.

The way in which gender-related variables are incorporated into statistical analysis depends on whether the research question requires precise relationships between outcomes and specific gender-related variables to be examined, or whether a composite gender score can be generated to reduce the number of variables within a statistical model. Statistical tests should be performed whether the gender-related variable is considered to have direct independent effects on the health outcome, and thus should be treated as a main effect, or whether it has indirect effects, and therefore should be included as a mediating factor (11). In addition, it is possible that a gender-related variable affects the direction or strength of the relationship between an independent variable and health outcome, in which case interactions should be examined. This could include interaction terms between sex and gender-related variables to determine whether conforming to gender norms differentially impacts men or women. When lacking statistical power for detecting such interactions, subgroup analyses using sex and/or gender-related variables can be used, albeit limited to descriptive purposes.

#### Retrospectively incorporating gender into cardiovascular research studies.

In 2019, the Gender Outcomes INternational Group: to Further Well-being Development (GOING-FWD) Consortium underwent the nearly year-long process of developing the GOING-FWD methodology to retrospectively assess the associations of gender-related factors with health outcomes (31). This methodology advocates strongly for the development of a multidisciplinary team and merged data sets to harness big data to overcome samples size issues inherent to studying gender.

**Figure 1.**
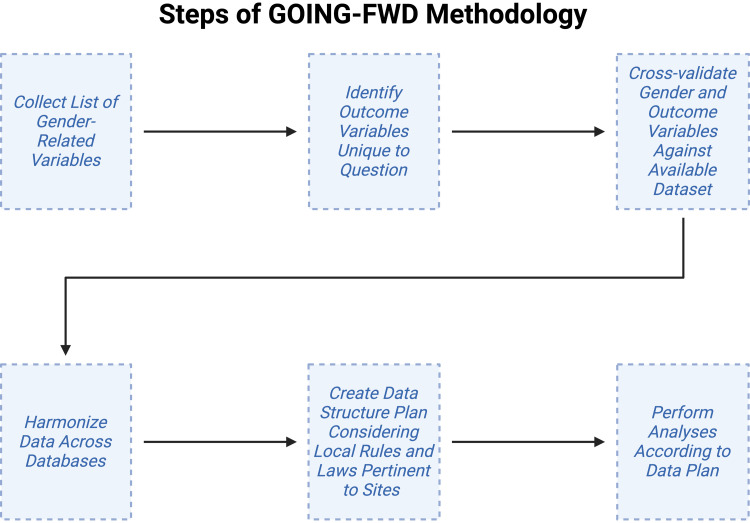
The steps of the Gender Outcomes INternational Group: to Further Well-being Development (GOING-FWD) methodology, starting with collecting a list of gender-related variables and ending with performing data analysis according to a predetermined plan. Figure created with a licensed version of BioRender.com.

Figure 1 summarizes steps of the GOING-FWD methodology (31). To start at *step 1*, the Consortium developed a wish list of gender-related variables to extract from data sets. This wish list serves as a standardized tool for all researchers seeking a refined list of gender-related variables. *Step 2* of the GOING-FWD methodology involves the identification of outcome variables unique to the research question of interest. In *step 3*, both outcome and gender-related variable lists should be rescreened and cross-validated by participating centers based on availability within databases. In *step 4*, data are harmonized across databases. This is a critical step in big data analysis, as it aims to minimize deviations in measurement across databases, which have been presumably generated independently. Finally, the Consortium found the need for the creation of a data structure, which was necessary given regional regulations involving health data. For example, if data were not transferable between centers even when anonymized, the data structure plan stated that individual analyses would be performed locally, generating data that could then be included in a meta-analysis of data from all participating centers. Indeed, this is a limitation of the big data approach, as the Consortium highlighted that data accessibility and protection issues between jurisdictions could limit its use. It is recommended that privacy-enhancing technologies be used to permit analyses without requiring the transfer of personal health information.

The GOING-FWD Consortium recommended that authors wishing to retrospectively study the impact of gender-related variables on health outcomes include the creation of a multidisciplinary team including gender experts and patient partners. This also applies to data harmonization, which is a time-consuming process susceptible to pitfalls related to reductions in data quality and thus necessitates individuals with technical skills related to computer science and mathematics. Finally, the Consortium noted that the lack of standardized definition of gender-related factors has been perceived by researchers as an obstacle to conducting research into the impact of gender on health outcomes, even by those who are interested in the topic. The authors strongly encourage clinical and preclinical researchers to start from what they have, even if only one gender-related factor is available.

One caveat is the difficulty in assessing the effects of gender-related variables on cardiovascular outcomes in small cohort studies. For example, compared with large observational studies, group sample sizes in cross-sectional and experimental cardiovascular physiology studies are typically small (i.e., 10–30 participants), which likely limits their ability to account for gender-related variables. Therefore, tools to assess gender and gender-related variables in small cohorts require development and then refinement over time as gender norms change. This is an ongoing area of research that requires further consideration, discussion, and examination from the human cardiovascular physiology community moving forward.

## INCORPORATING SEX IN ANIMAL STUDIES

### Considerations for Study Design

Considering sex as a biological variable necessitates that the default is that both sexes will be used unless there is strong scientific reasoning to include only one sex. Examples of strong rationale for not including both sexes would be examining processes that only affect one sex such as cardiovascular complications of pregnancy or testing the effects of prostate cancer therapies on cardiovascular health. A natural sex-biased ratio of disease incidence is not sufficient to justify a single sex selection study by default. Instead, this effect could be capitalized on and incorporated into both the experimental design and interpretation of results, as evaluating differences in pathology or response between sexes may uncover the mechanisms benefiting the protected sex. The rationalization that male animals have historically been used, creating an obstacle for comparing results to past findings, is totally insufficient and instead strengthens the reasoning for including females in studies. Stating that female animals will be used in the future, as noted previously regarding the inclusion of women in studies, is similarly not a satisfactory rationale as time has shown. Data have disproven initial concerns that female mice exhibit higher variability in physiological traits, negating another common argument against sex inclusion; in fact, female mice often exhibit lower standard deviations than males across a variety of anatomical, behavior, and physiologic traits (100–103). Therefore, including female animals can improve the power of the study. Cage effect variability due to dominance, fighting, and environmental acoustics is greater in males than females and contributes significantly more toward intra/intergroup variation (104). For these reasons, caging conditions, breeding status (e.g., sex naïve males/females vs. retired breeders), and caging numbers should be considered in experimental design. In addition, if a study requires maintenance of an in-house mouse breeding colony, there is an ethical responsibility to ensure both sexes are responsibly used to avoid the unnecessary generation of animals (105, 106). SABV is especially important when evaluating aging effects on cardiovascular disease (CVD) in older animals (107–112).

#### Using models of sex hormone regulation.

Although many researchers whose focus is to evaluate sex differences in disease pathology use models such as gonadectomy that test sex hormone regulation, the use of both sexes does not necessitate taking measures to an extreme. Reporting of estrous cycle phase or requiring ovariectomy/orchidectomy is often not necessary. Moreover, hormone supplementation is not essential unless the proposed mechanism implies sex hormones are regulating the pathway of interest. Even then, researchers should be mindful of the complex physiological changes that are associated with hormones. For example, physiologically, estrogens are not continuously elevated or maintained at constant concentrations, and thus continuous infusion of estrogens by osmotic pump may not be the best method for hormone supplementation. The lack of data or use of particular procedures is not valid rationale for exclusion of a particular sex. In fact, the editorial board of *AJP-Heart and Circ* does not necessarily require authors to uncover the underlying mechanism(s) when sex differences are first identified. The SABV policy does not necessitate that all studies be sufficiently powered to probe specifically for sex differences; instead, the policy requires only that consideration be given to the possible impact sex could have on results, interpretations, and conclusions.

If the study aims to address the influence of sex hormones, one must survey which model best addresses the question. Rodents do not experience a natural menopause and thus models such as ovariectomy or ovitoxins including diepoxide form of 4-vinylcyclohexene can be used to test the influence of female sex hormones (101, 113–115). The ovariectomy model is easily reproducible and has been used extensively to dissect out mechanisms of female hormones. In a recent study by the Merryman group (116), demonstrated with bilateral ovariectomy, female mice on a Western diet displayed left ventricle hypertrophy without concurrent fibrosis or aortic valve stenosis. This novel model did not require genetic manipulation or drug treatment, closely representing the aging process of postmenopausal women. A limitation of the ovariectomy model is that the rapid loss of ovarian hormones postsurgery does not mirror physiological menopause implying another model may be best if identifying menopausal mechanisms is the purpose of the study (115, 117). The 4-vinylcyclohexene model chemically induces follicular loss resulting in longer cycles, fluctuations followed by an eventual drop in estrogen, and increased follicular stimulating hormone mimicking perimenopause in humans (113, 114, 118). Although early studies indicated a potential toxic and carcinogenic effect of 4-vinylcyclohexene, histopathological evaluation at 6 mo and after 15 initial daily doses of 4-vinylcyclohexene (160 mg/kg/day) revealed that the adverse effects were limited to the reproductive tract with no pathological effects observed in uteri, kidneys, adrenals, spleen, liver, lung, heart, brain, intestine, or pituitary tissues (117). This dose is considerably less (980-fold) than that observed to induce any toxic or carcinogenic effects (119, 120). To evaluate estrogen downstream signaling, models of G protein-coupled estrogen receptor (GPER) or estrogen receptor (ER)α/β-deletion or inhibition can be used (112, 121, 122). Using a GPER inhibitor, Manning et al. (121) identified GCN5L1 as a potential mediator of divergent cardiac mitochondrial function between men and women.

#### Effects of testosterone on cardiovascular physiology.

As discussed in the clinical section of these guidelines, much of the data illustrating the effects of testosterone on physiological systems are conflicting. The action of testosterone is complex in part due to aromatization of testosterone to estradiol and the fact that testosterone declines with age (123, 124). This conversion may explain some of the contradictory results observed in animal studies. The most common animal model to test for the role of male sex hormones is surgical castration or bilateral orchiectomy. Following surgical castration, circulating testosterone drastically decreases (125). A strength of this model is that a range of testosterone can be administered back, enabling researchers to test the effect of low versus high testosterone levels, and mimicking intraindividual variability found in men. Although not used as extensively as surgical castration, chemical or pharmacological castration via gonadotropin-releasing hormone analogs such as degarelix or leuprolide has been used to suppress testosterone and study the effects on CVD (126). This model represses testosterone by inhibiting the pituitary gland from releasing luteinizing hormone (127). Mouse models deleting or mutating the androgen receptor are useful for dissecting the effect of downstream testosterone signaling in CVD. The testicular feminized mouse model is achieved by an X-linked, single base-pair deletion in the gene encoding the androgen receptor to result in low circulating testosterone concentrations and a nonfunctional androgen receptor (128). Using models of androgen receptor deficiency, the testicular feminized mouse, and androgen receptor deletion mouse models, two separate studies distinguished androgen receptor-dependent mechanisms from effects because of aromatization of testosterone to estradiol in a mouse model of atherosclerosis (129–131). In summary, there are many models available and more in development to test the effects of sex hormones, each with its own technical demands and limitations for application.

#### Models of sex chromosomal effects.

Although sex hormones have historically been implicated as a dominant factor driving the differences observed between the sexes, more recent studies are pointing to sex chromosomes as likely regulators for cardiovascular pathophysiology (132–134). Although these early studies reveal possible effects of sex chromosomes, additional studies are needed to identify specific X and Y genes that influence cardiovascular disease in a sex-biased manner.

The most common animal model used to evaluate the effects of sex chromosomes on CVD pathology is the four-core genotypes (FCG) mouse model. The FCG mouse is used to determine the effects of sex chromosome complement (XX vs. XY) by removing the testis-determining gene *Sry* and placing it on an autosome resulting in a gonadal-type model not controlled by the sex chromosomes (135). The FCG model allows dissection of phenotypic differences caused by sex chromosome in addition to the differential effects of gonadal hormones. Future studies are needed to determine how sex chromosomes are affected (i.e., synergized or antagonized) by sex-specific hormonal environments.

In addition to sex-specific transgenic models, certain genotypes and mouse strains influence sex differences in disease progression. Sex-related differences in models of CVD have been observed in several genotypes/strains (136–139). For example, in a mouse model lacking natriuretic peptide receptor A, sudden death occurred in 100% of male mice but only in 6% of female mice (136). Conversely, in a model of cardiac-specific overexpression of the β_1_-adrenergic receptor, female mice died at an earlier age (7.0 ± 0.7 mo of age) compared with males (12.4 ± 0.7 mo of age) (140). Similarly, in a platelet-derived growth factor-C overexpression model, male mice developed a hypertrophic phenotype, whereas female mice had a more severe phenotype developing dilated cardiomyopathy that led to heart failure and sudden death (141). The sex dimorphisms observed were not due to differences in the level of transgene expression indicating observed distinctions are likely due to downstream sex-dependent signaling.

### Considerations for Data Collection

Data analysis should minimize the use of assumptions. For example, it is best to not assume sex differences exist or do not exist; it is best to test the assumption. Another assumption that should be tested is that because both sexes display a similar terminal pathology, there are no differences in the molecular pathways invoked. There are frequent reports in the literature of similar end point physiological outputs resulting from sex-dependent differences in molecular and cellular responses and activated pathway(s) (142–147). As one example, combined animal and clinical data sets reveal that after myocardial infarction, both aged men or male mice upregulate the liver X receptor (LXR)/retinoid X receptor (RXR) pathway whereas aged women or female mice are desensitized to the LXR/RXR pathway. Regardless of this difference in signaling, all groups arrive at a similar pathophysiological end point and show comparable signs of heart failure (142). As a second example, assessment of the drug 5-aminoimidazole-4-carboxamide riboside (AICAR), an AMP-activated protein kinase agonist, on age-induced diastolic dysfunction was found to attenuate the age-related increase in left atrial volume in female but not in male mice because of a blunted response to AICAR and decreased AMP-activated protein kinase phosphorylation in males (146). Both of these examples highlight that sex-specific mechanisms in disease pathology can affect the efficacy of potential interventions and should be taken into consideration when analyzing the data.

An important way to fully understand study results is to record as many primary and secondary outcomes for all samples collected. For example, even if no overt sex differences are observed under pathological or control settings, differences may be observed in response to specific treatments, especially when G protein-coupled receptors or other hormone-related receptors are involved (112, 121, 148–152). Spironolactone, a diuretic used to treat patients with heart failure in the Treatment of Preserved Cardiac Function Heart Failure with an Aldosterone Antagonist (TOPCAT) trial was associated with reduced all-cause mortality in women (hazard ratio, 0.66; *P* = 0.01), but not in men (hazard ratio, 1.06; *P* = 0.68) with a significant sex-effect interaction (*P* interaction = 0.02) (153). In addition to potential differences in direct signaling pathways, the difference was also amplified by a higher study withdrawal rate in men because of increased incidences of gynecomastia and hyperkalemia as side effects. Similarly, calcium channel blockers induce peripheral edema in women, potentially leading to decreased adherence and drug discontinuation (154). Thus, both direct and indirect molecular mechanisms can be differentiated by sex/gender responses to therapy even when the end phenotype is similar. As such, data collection consideration should be given when designing experiments, to ensure that full responses at molecular, cellular, and organ levels are evaluated.

### Considerations for Data Analysis, Reporting, and Interpretation for Animal-Based Studies

The first consideration involves determining sample sizes and performing a power analysis. Although the SABV policy states that both sexes should be included unless strong scientific reasoning to include only one sex, it does not mandate researchers to capriciously double sample sizes to accommodate the addition of both sexes. There is also no requirement for studies to be sufficiently powered to probe specifically for sex differences. Recently, a working group of extramural experts involved in sex differences research came up with the *n* + 2 concept (155). This working group advised that adding just two additional animals (*n* + 2) will have the same power as a single-sex study with a sample size of *n*. To obtain the same residual degrees of freedom in a two-way ANOVA that includes sex as a factor, only two additional animals were needed for a sufficiently powered study. In some instances, *n* + 2 may be sufficient to identify a trend just not to determine significance. On these occasions, a larger sample size may be needed for a particular parameter. Similarly, a series of sample size analyses run by Buch et al. (156) found that total sample size increased by a maximum of 33% when including data analysis by sex.

#### Separating by sex for analysis.

There is a lack of consensus on the best way to analyze data when both sexes are used. Although using both sexes in a combined analysis will allow identification of pathways and targets that affect both sexes in combination, the best way to formulate a more tailored therapeutic approach for each sex may entail identifying similarities and differences across sexes (157). The most common way to assess differences between sexes is a two-way ANOVA which provides tests for both main effects (treatment and sex) as well as a test of interaction.

It is also common practice to separate data and evaluate each sex individually, provided the study is powered sufficiently to allow analysis by sex. The use of this approach revealed a novel role for sex-dependent neutrophil responses to myocardial infarction (142). Normalizing response to baseline data for respective sex highlighted the sex-specific response. This strategy removes the effect of differences that may be observed at baseline or between controls to focus on differences in pathological responses between sexes.

The approach to divide into groups based on sex used should be considered carefully, as it can result in a loss of power and consequent failure to detect an effect that is truly present in one or both sexes (false negative). Even if there is no a priori rationale to segregate or bias to one sex, the aggregated data should be reported so that the sex of each individual is clearly indicated. Designating the data for each individual sex by color or shape will make it easier for the reader to interpret the data for potential future studies. An important consideration is the critical need to define plans for statistical evaluation of two-sex studies before the study is performed.

In summary, Table 2 provides recommendations for considering SABV in animal studies. We recommend authors use both sexes unless there is a clear scientific rationale for using only one sex; we will also clearly state that there are very few examples where this rationale exists. The use of female animals does not necessitate reporting of estrous cycle phase or require gonadectomy and does not require that the mechanism for observed differences be presented. For studies dissecting potential sex differences, the study design should be considered carefully, acknowledging limitations in the applicability of findings that may arise from the animal model, methods, and analyses used.

**Table 2.** Minimum and optimal recommendations for consideration of sex as a variable in animal studies

Minimum	Optimal
*Study design, data collection, and data analysis*	
• Power analysis to determine feasibility of including both sexes. • Consider level of evaluation (molecular, cellular, organ, and whole animal). • Animal housing (e.g., cage conditions, group/single housing). • Breeding status or history (i.e., virgin, bred, or retired breeders). • Collect primary outcomes for both sexes.	Minimum, plus: • Sample size sufficient for analysis within and between sexes. • Match animals for breeding history. • Estrus cycle and/or hormone levels. • Collect and analyze secondary outcomes for both sexes. • Analyze data within and between sexes.
*Reporting*	
• Report a priori power analysis results if primary focus is on sex differences. • Present collective results as well as data from each sex individually. • Include general breeding history of animals. • Effects unique and common to sexes (similarities are as important as differences).	Minimum, plus: • Statistical analysis of collective and sex-specific data for comparison between sexes. • Report sex hormone concentrations or estrus cycle information.
*Data interpretation*	
• Distinguish statistical vs. biological differences. • Include limitations (e.g., sample size, confounding variables like breeding history). • Keep focus within the context of your sample set.	Minimum, plus: • Include interpretation of data based on estrus phase and sex hormone levels as applicable.

## INCORPORATING SEX AND GENDER IN CELL-BASED STUDIES

### Considerations for Study Design

Sex omission and male bias are still widespread in cell experiments (21), and researchers should consider using established cell lines of both sexes to assess the impact of sex-linked genes on phenotype and functional responses. Authentication of cell line sex should be an integral and routine part of scientific practice since such is recommended or mandated by many funding agencies. The American Type Culture Collection (ATCC) recommends cell authentication when a cell line is acquired, after 10 passages, after preparing a cell bank, or when in doubt. ATCC and other cell lines (e.g., European Collection of Authenticated Cell Cultures and Japanese Collection of Research Bioresources Cell Bank) strive to report the sex of human cell lines using either the sex of the isolate subject or analyses of short tandem repeats (STRs) within the amelogenin genes AMELX and AMELY, which are on X and Y chromosomes, respectively. In some cases, STR analyses of alternative genes, such as DYS319, may be required because of preexisting mutations (including deletions) in X or Y chromosomes or genetic drift following extensive passaging. Research laboratory-based STR authentication can be executed using commercially available kits (e.g., Promega GenePrint 10 and GenePrint 24) or custom-designed primers (158), comparing the STR profile with reference data at the Cellosaurus resource on the ExPASy server (https://web.expasy.org/cellosaurus/) (159). However, using a commercial service provider is likely to be more reliable and time efficient. Although authentication of human cell line sex has become widely available and more routine, the sex of animal cell lines remains underreported even by cell repositories, often requiring additional effort on the part of investigators. Given the importance of SABV, that effort is necessary.

### Considerations for Data Collection

When the study design involves primary cells passaged in vitro or analyzed immediately ex vivo, sex authentication may be based on that of the cell/tissue donor. If the donor information is not available, STR authentication may fill this gap. Similar to human participant and animal studies, investigators should use donors of both sexes when designing in vitro studies, or at least describe why it is not possible to do so, thereby justifying any bias. When primary cells are used, it is also important to consider the influence of gonadal hormones to which they were exposed to prior isolation. Specifying the donor history (menstrual cycle, history of hormone replacement, or gender affirming therapies), if available, may at least allow estimation of gonadal hormone influences. When the donor history is not available, study design may include analyses of associated blood or urine samples. Alternatively, gonadal hormone exposure biomarker detection could be included in the study design.

Exposure of cells to gonadal hormones and hormone-like substances during in vitro culture may also introduce experimental bias with consequential misinterpretation of the results. Therefore, study design and data collection should include such a bias risk assessment, recording serum levels of gonadal hormones in the culture media, as well as concentration of the pH indicator phenol red, which is widely known to have estrogen-like effects.

### Considerations for Analysis, Reporting, and Interpretation

If the cell line sex has been determined, this should be reported in the manuscript accordingly. If the cell line sex has not been determined, it should be described in the study limitation section. If cell lines of both sexes were used, the experimental data should be reported separately with attention given to the sex differences. When possible, gonadal hormone exposure before (donor serum levels or history of exposure) and during (concentration in the culture medium) the studies should be reported. Similarly, exposure to hormone-like substances or efforts to minimize the exposure should be reported to increase study reproducibility. If the information is not available, it should be stated clearly in methods or in the study limitations.

Study results and conclusions would be stronger if attention is given to sex-dependent parameters. The potential impacts of sex-linked genetic and/or epigenetic factors and environmental exposure to gonadal hormones and hormone-like substances should be assessed and discussed when possible. Furthermore, the interpretation of gonadal hormone effects on cardiovascular cell studies can be confounded by differential responses of genetically XX- versus XY-derived cells. The biology behind these differences is frequently poorly understood. Establishing clear-cut differences in carefully designed studies analyzing cells derived from donors of both sexes, separately, can lay the foundation for additional mechanistic studies defining molecular determinants of these differential responses. This can lead to paradigm-changing understanding in SABV and, in turn, gender-specific therapeutic approaches in a wide array of pathologic states.

In summary, Table 3 provides recommendations for considering sex in cell-based studies.

**Table 3.** Summary of recommendations for inclusion of SABV in cell-based studies: the 4 Cs

Concept	Recommendations
Consider	• Sex-linked genetic, epigenetic, and environmental factors in study design to include SABV.
Collect	• Short tandem repeat analysis or other data on cell line sex and hormonal exposure.
Characterize	• Effects of cell line sex and environmental exposure on results.
Communicate	• Results of cell line sex authentication in materials and methods, effects of cell line sex in results and discussion, insufficient information on cell line sex in discussion or *Limitations of the Study*.

“NIH policy on sex as a biological variable” (SABV) may be found at https://orwh.od.nih.gov/sex-gender/nih-policy-sex-biological-variable.

## CONCLUSIONS

The historical failure to consider sex and gender variables in research, and to discriminate against the inclusion of women or female animals for scientifically unsound reasons, comes at a cost. Knowledge about sex differences and the role of gender in health and disease was lost, and gains in treatment, diagnosis, and management of disease have not been equally realized across sexes and genders. By comparison, women and girls have lagged behind improvements in health outcomes relative to men and boys, and those who do not identify with the binary sex and gender classification system often used in research have been largely ignored. The goals of biomedical and health research are to improve the health of all communities and provide greater opportunities for prolonged life span. The exclusion or omission of groups based on sex or gender serves as an obstacle to these goals; it creates a two-tiered system of research and healthcare that disproportionately rewards some and disadvantages others. Including sex or gender as a research variable will not only improve the health of society, it lays a solid foundation of knowledge, to build future advances in science and health that benefit all.

**Figure 2.**
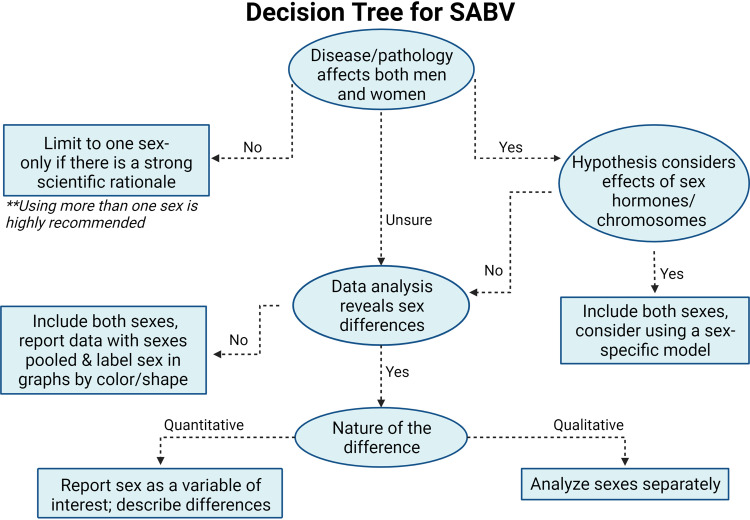
Decision tree for how to incorporate both sex and gender according to the provided guidelines. Figure created with a licensed version of BioRender.com. SABV, sex as a biological variable.

Including SABV in study design and data collection, analysis, and interpretation improves the rigor and reproducibility of a study and improves the likelihood of study results informing clinical decision-making and improving outcomes. The study design and methods used should be considered carefully, acknowledging limitations in the applicability of findings that may arise from the model, methods, and analyses used. We have provided a framework to facilitate incorporation of sex as a biological variable when collecting, analyzing, or reporting data (Fig. 2). In cardiovascular physiology, incorporating both sex and gender into human, animal, and cell studies is a necessary component of optimal study design. We acknowledge that to optimally integrate the recommended guidelines requires a significant amount of time and funding, and as such have also provided minimum requirements to help scientists as we all work toward achieving this goal.

## GRANTS

The authors acknowledge funding from National Institutes of Health Grants AI168968 and HL150766 (to J.R.B.); AR079591 (to J.R.B. and T.O.Y.); ARO84226 and DK124344 (to J.G.R.); GM151274 (to M.L.L.); AG062319, AG072094, and AG075544 (to K.L.M.); HL147998 and R15HL165325 (to A.T.R.); HL146558 (to M.M.W.); GM151274 (to M.L.L.); HL093056 and HL146833 (to B.A.H.); NR019054 (to Q.E.D. and B.A.H.); AR084221 (to Q.E.D.); and HL135790 (to W.D.M.) and from the Biomedical Laboratory Research and Development Service of the Veterans Affairs Office of Research and Development Grants I01BX000505 (to M.L.L.) and I01BX005848 (to K.Y.D.-P.). N.C. is funded by the Military Operational Medicine Research Program at the U.S. Army Medical Research and Development Command. L.P. and the GOING-FWD Consortium is funded by the GENDER-NET Plus ERA-NET Initiative under Project Ref. No. GNP-78: Canadian Institutes of Health Research Grant GNP-161904. C.W.U. is funded by the Natural Sciences and Engineering Research Council of Canada Discovery Grant RGPIN-2018-05961 and the Fonds de Recherche du Québec Santé (Chercheur-boursier Junior 1 268920), and W.G.P. is a Senior Career Investigator for Improving the Heart and Brain Health for Women in Canada (Heart and Stroke Foundation of Canada and Health Canada) and supported with a Heart and Stroke Foundation of Canada Grant-in-Aid G-21-0031543 and Natural Sciences and Engineering Research Council of Canada Discovery Grant RGPIN-2018-04732. J.G.R. is supported by the Ludeman Family Center for Women’s Health Research at the University of Colorado School of Medicine. K.Y.D.-P. acknowledges investigator-initiated research project funding from Merck and Co., Inc.

## DISCLAIMERS

The content is solely the responsibility of the author and does not necessarily represent the official views of any of the funding agencies.

## DISCLOSURES

Dr. Timur O. Yarovinsky reports consulting fees from CaroGen Corporation outside the submitted work. Dr. Jeffrey R. Bender reports consulting fees from Pfizer and Esperion unrelated to the submitted work. Dr. Megan M. Wenner is a consultant for Orchestra BioMed outside the submitted work. Dr. Nisha Charkoudian is an employee of the U.S. Army. The opinions or assertions contained herein are the private views of the author(s) and are not to be construed as official or as reflecting the views of the U.S. Army or the Department of Defense. Citations of commercial organizations and trade names in this report do not constitute an official Department of the Army endorsement or approval of the products or services of these organizations. None of the other authors has any conflicts of interest, financial or otherwise, to disclose.

Merry Lindsey is an editor of *American Journal of Physiology-Heart and Circulatory Physiology* and was not involved and did not have access to information regarding the peer-review process or final disposition of this article. An alternate editor oversaw the peer-review and decision-making process for this article.

## AUTHOR CONTRIBUTIONS

C.W.U., M.L.L., A.T.R., B.A.H., C.E.T., W.D.M., D.K., J.R.B., J.G.R., K.L.M., L.P., M.M.W., M.O., T.O.Y., N.S.S., N.C., Q.E.D., J.D.M.-B., W.G.P., and K.Y.D.-P. conceived and designed research; J.D.M.-B. and K.Y.D.-P. prepared figures; C.W.U., M.L.L., A.T.R., B.A.H., C.E.T., W.D.M., D.K., J.R.B., J.G.R. K.L.M., L.P., M.M.W., M.O., T.O.Y., N.S.S., N.C., Q.E.D., J.D.M.-B., W.G.P., and K.Y.D.-P. drafted manuscript; C.W.U., M.L.L., A.T.R., B.A.H., C.E.T., W.D.M., D.K., J.R.B., J.G.R., K.L.M., L.P., M.M.W., M.O., T.O.Y., N.S.S., N.C., Q.E.D., J.D.M.-B., W.G.P., K.Y.D.-P. edited and revised manuscript; C.W.U., M.L.L., A.T.R., B.A.H., C.E.T., W.D.M., D.K., J.R.B., J.G.R., K.L.M., L.P., M.M.W., M.O., T.O.Y., N.S.S., N.C., Q.E.D., J.D.M.-B., W.G.P., and K.Y.D.-P. approved final version of manuscript.
